# Supplementary material for: Advancing Exoskeleton Development: Validation of a Robotic Surrogate to Measure Tibial Strain
Source: Bioengineering (Basel). 2024 May 15;11(5):490. doi: 10.3390/bioengineering11050490 (PMC11118925; doi:10.3390/bioengineering11050490)
Supplement: Supplementary file 1 [file bioengineering-11-00490-s001.zip › bioengineering-2965453-supplementary.pdf]

bioengineering

Supplementary Materials

Robotic Surrogate Fabrication Manual

Materials

- Strain Sensors: Five 45 deg strain gauge rosettes, adhesive, Teflon tape, butyl rubber, cable cover wrap, and RJ45 connectors
- Force Sensor: Linear XDCR, endpiece cable attachments, break-out tube with end caps
- 3D printed Bones Rigid 10K: Tibia, Fibula, Patella, femur, and composite foot
- Ankle Joint: Distal and proximal aluminum components
- Steel calcaneus insert, clevis, and cotter pin
- Encoder Assembly: Encoder, custom encoder bracket, DB9 Male breakout connector
- Bowden cable: Inner Vectran cable and outer sheath
- 5/8" diameter and 3\8" diameter steel sections
- 2 Bronze Bushings
- Several lengths of ¼-20 bolts and locknuts
- Pyramid female to Rod connector with metal sleeve insert
- Fiberglass wrap
- Dragon skin silicone
- Stockings
- Foam wedge
- Epoxy
- Putty
- Electrical tape
- Plastic wrap
- 50th percentile male leg mold
- Duct tape

Tools

- Screwdriver
- Dremel
- Box cutter
- Shears
- Sandpaper on block
- Bandsaw
- Table Vice
- Drill and drill bits
- Mill and Lathe
- Table Saw

Assembly of Ankle/Tibia/Femur

1. Insert a length of 5/8″ diameter steel into the proximal axial hole of the femur and adhere with epoxy.
2. Use a foam wedge to align the femur relative to the tibia and adhere with epoxy.
3. Secure the patella to the knee joint in the proper anatomical location with epoxy.
4. Reinforce the knee joint and patella with a generous amount of epoxy.
5. Use epoxy to adhere the short length of Bowden sheath inside of the Bowden cable conduit on the posterior side of the tibia with the cable passing through the designated hole on the posterior side of the tibia. Ensure that the epoxy is only in contact with the outside of the outer sheath as not to interfere with functionality.
6. Pass a sufficient length of the Vectran cable through the Bowden sheath to complete the Bowden assembly.
7. On the distal end of the tibia, tap the distal axial hole for ¼″-20 thread.
8. Secure the proximal ankle joint component with a ¼″-20 bolt into the axial hole of the tibia and align the medial facets.
9. Fill in the gap between the tibia and metal ankle component with a generous amount of epoxy.
10. Press fit the two brass bearings into the proximal aluminum ankle where the axel will be inserted. (See Figure S1)

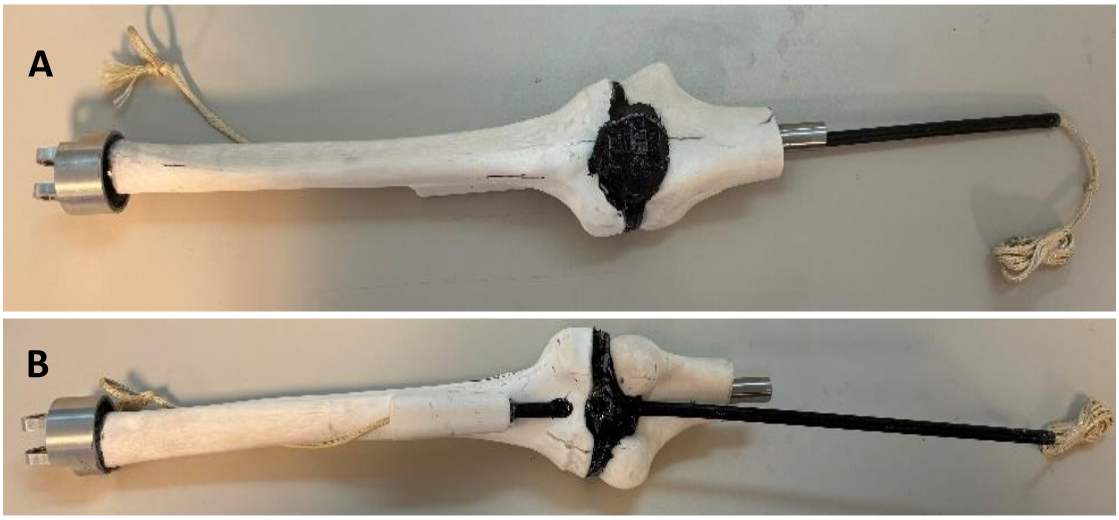

Figure S1: (A) anterior and (B) posterior views of aligned knee assembly.

11. Place the fibular head on tibia in the correct anatomical location and secure with epoxy.
12. Mark the intended locations on the tibia of the five-strain sensor such that the fiberglass wrap in the following step does not obscure sensorization:
    a. MedDist: Medial surface at 17.5% length from ankle
    b. MedMid: Medial surface at 50% length
    c. LatMid: Lateral surface at 50% length
    d. PostMid: Posterior surface at 50% length
    e. MedProx: Medial surface at 82.5% length from ankle
13. Wrap the knee joint in the moisture activated white fiberglass wrap to reinforce the knee joint. Allow sufficient time to completely set.

14. Clamp the pyramid connector and sleeve to the femur pylon. (See Figure S2)

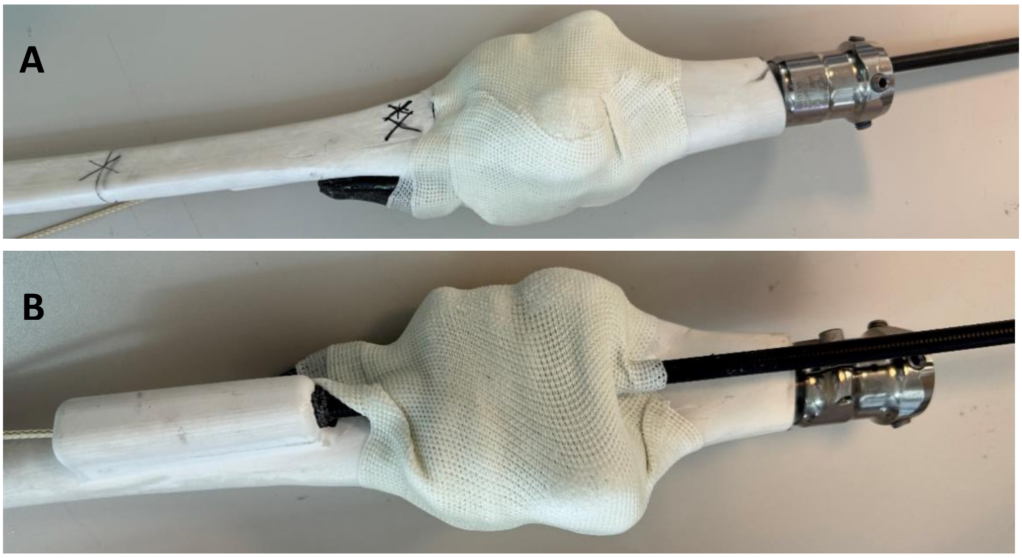

Figure S2: (A) anterior and (B) posterior views of completed knee assembly.

Assembly of Foot/Ankle

1. Grind down the surfaces of the composite foot bone as needed to accommodate the distal ankle joint component and tap the two heel holes with ¼″-20 thread.
2. Secure the distal ankle joint component to the composite foot bone with a vertical ¼″-20 bolt and a medial to lateral ¼″-20 bolt and secure both with vibration resistant nuts.
3. Secure the heel insert into the heel and secure with the two ¼″-20 bolts. Insert the clevis and cotter pin into the calcaneus of the composite foot. (See Figure S3)

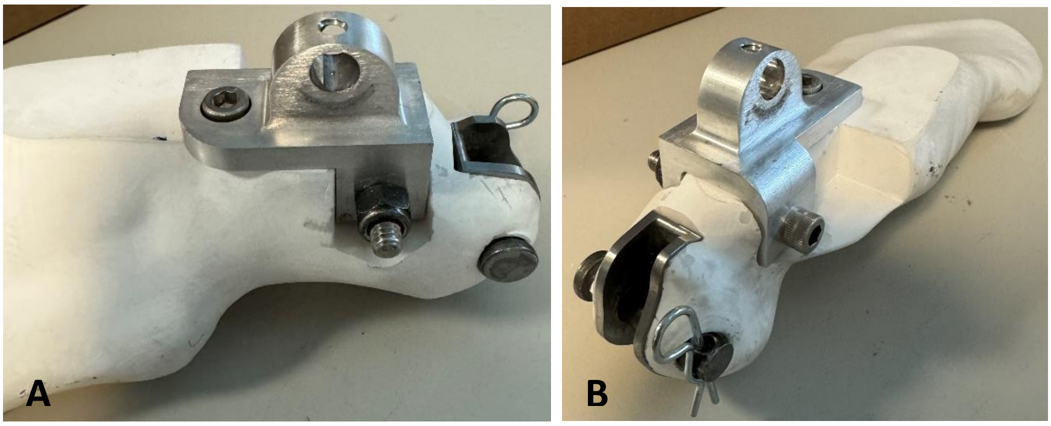

Figure S3: (A) lateral and (B) posterior/medial views of foot assembly.

4. Assemble the ankle joint with encoder, encoder bracket, and 3/8″ diameter steel axel. This will need to be done carefully with small screws and set screws. One screw to secure the bracket to the tibial bone where exposed inside the aluminum ankle component, two small screws to secure the bracket to encoder casing, and two set screws to tighten the inner encoder collar around the ankle joint axel.

Strain Sensor Application on Tibia

1. Adhere the five 45 deg strain gauge rosette sensors to the following locations as per the rosette installation instructions provided by VPG Inc.:
    a. MedMid: Medial surface at 50% length
    b. MedDist: Medial surface at 17.5% length from ankle
    c. MedProx: Medial surface at 82.5% length from ankle
    d. LatMid: Lateral surface at 50% length
    e. PostMid: Posterior surface at 50% length
2. Attach the RJ45 to screw terminal break out connectors to the rosette wires.
3. Ensure that all strain gauge rosettes are read properly by acquisition system before proceeding to the next step.
4. Cover each sensor with a butyl rubber patch and wrap in Teflon tape.
5. Organize and protect the wires with an abrasion resistant braided sleeve. (See Figure S4)

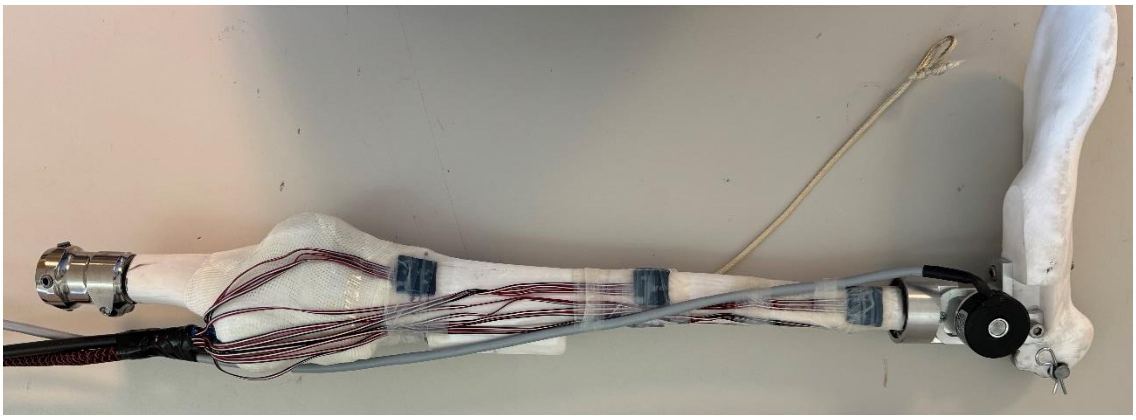

Figure S4: medial view of completed and sensorized musculoskeletal apparatus.

Pouring Silicone

1. Prior to pouring the silicone, form putty around the lower tibia, mid-foot, and heel to form the outer edges of the joint capsule. To form the join capsule, wrap plastic wrap all around the ankle joint leaving the forefoot, mid tibia, and heel uncovered.
2. Utilizing electrical tape, wrap the outside edges of the wrapped area, over the putty locations, to seal off the ankle joint. (See Figure S5A)

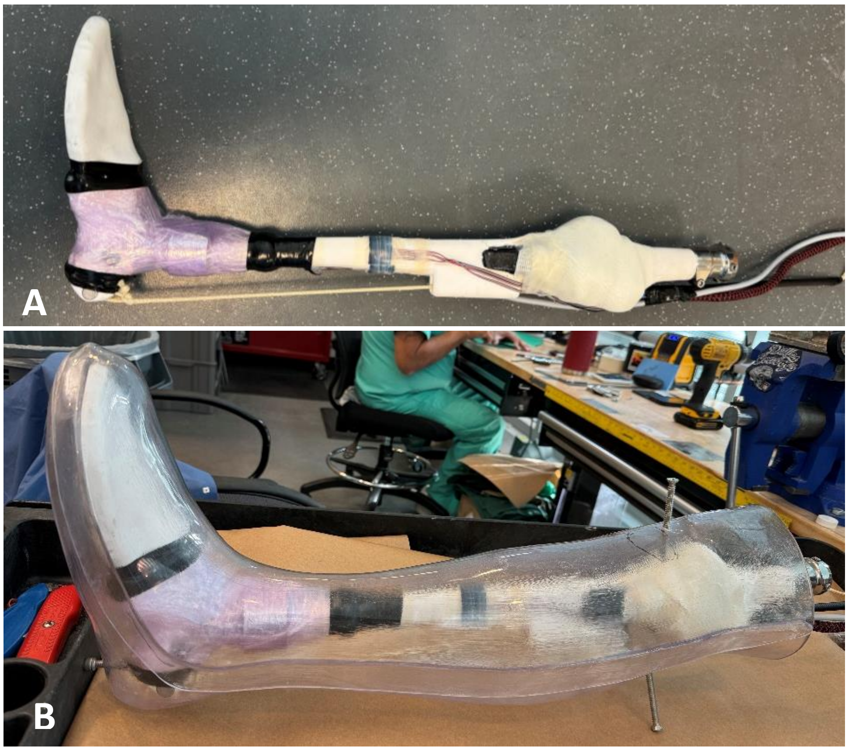

Figure S5: (A) lateral view of completed musculoskeletal apparatus with joint capsule and (B) musculoskeletal apparatus in the 50th percentile male leg mold with pin alignment prior to silicone pour.

3. Coat the Bowden inner cable in Vaseline to prevent adhesion to the silicone during the setting process.
4. Place the Bowden cable under tension and fix the skeleton inside the leg mold with screw pins. (See Figure S5B)
5. Seal the mold with duct tape to prevent leaking. Cover the sensor cords to protect from the liquid silicone.
6. Mix the silicone liquid with 40% thinner then pour into the mold and allow to set for at least 24 hours in an upright position. (See Figure S6A)
7. Pull the leg from the mold, trim off excess silicone, and cover in two layers of stocking. (See Figure S6B)

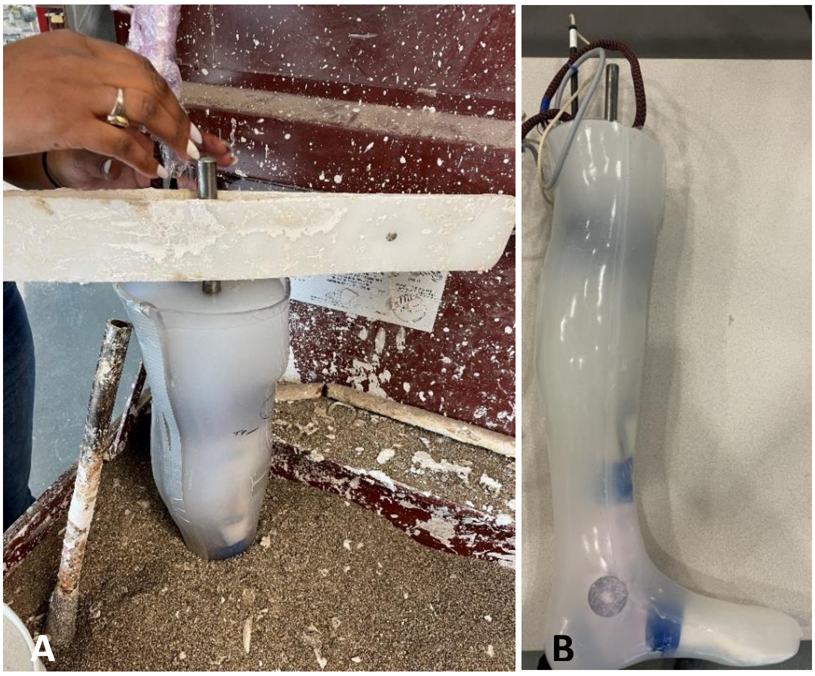

Figure S6: (A) mold with poured silicone stabilized to allow for silicone to set and (B) the completed robotic surrogate.

Bowden Cable

1. The force sensor has an endpiece screwed on to each threaded end to allow for bowline knot interface with the Bowden assembly inner cable.
2. This assembly is encased in the breakout tube which interfaces with set screws with the outer Bowden Cable sheath (See Figure S7).

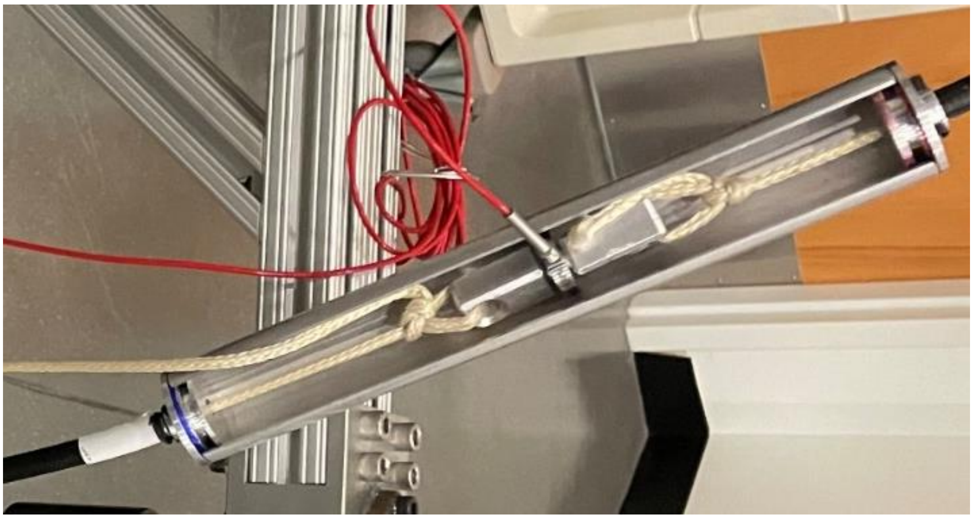

Figure S7: The breakout tube of the Bowden cable assembly instrumented with a force sensor to allow for force feedback control of the robotic surrogate Achilles tendon.
